# Exercise Improves Spatial Learning and Memory Performance through the Central GLP-1 Receptors

**DOI:** 10.1155/2022/2900628

**Published:** 2022-06-21

**Authors:** Majid Taati, Peyman Esmaeili Fard Barzegar, Abbas Raisi

**Affiliations:** ^1^Department of Physiology, Faculty of Veterinary Medicine, Lorestan University, Khorramabad, Iran; ^2^Department of Clinical Sciences, Faculty of Veterinary Medicine, Lorestan University, Khorramabad, Iran

## Abstract

The glucagon-like peptide 1 (GLP-1) is a hormone which is produced in the enteroendocrine L-cells in the ileum and the neurons of nucleus tractus solitarius (NTS) in the brain which has numerous metabolic effects. The central GLP-1R's role in cognitive functioning is well known. On the contrary, it has been shown that exercise has positive effects on brain function. So, we decided to elucidate whether the central GLP-1 has a role in memory and learning. Thirty-two rats were used in this experiment in 4 groups. After anesthetizing the rats, the right lateral ventricle was detected, and a cannula was directed to the ventricle. Ten micrograms of exendin-3 or sterile saline, according to the group, was injected via ICV once daily for seven days. The rats in the exercise group considered an exercise period of one hour each day (17 meters per minute) for seven consecutive days. To evaluate the performance of memory and learning, a standard Morris water maze (MWM) tank was utilized. According to the results, the TE-exendin group showed a statistically significant difference from the TE-SAL group in both parameters of latency and time in the zone. In summary, memory and learning were improved by GLP-1R in the exercise group, but not in the sedentary group, which we can hypothesize that exercise can affect memory and learning through this pathway.

## 1. Introduction

Memory and learning loss is regarded as one of the world's greatest issues brought on by age, accidents, and even some pharmaceuticals. Although several research have been conducted on medicines to prevent age-related cognitive decline, effective treatments for cognition and memory enhancement are not yet available (Coppi et al. [1]). The glucagon-like peptide 1 (GLP-1) is mainly composed of 160-amino acid proglucagon precursor protein (Bell et al. [2]; Ye et al. [3]) in the enteroendocrine L-cells found in the ileum of distal small intestine and large intestine. This hormone has numerous metabolic effects, such as decreasing stomach emptying, lowering the appetite, and glucose-dependent stimulation of insulin secretion. Moreover, GLP-1 protects neurons and decreases inflammation and apoptosis (Müller et al., [4]). GLP-1 is also known to originate in the brain, where it functions as a neurotransmitter (Paternoster and Falasca [5]). GLP-1 is released by neurons of the nucleus tractus solitarius (NTS) of the brainstem, hypothalamus, and cortical brain regions, which also express GLP-1 receptors (GLP-1R) (Llewellyn-Smith et al., [6]). The biological actions of GLP-1 are mediated by a G-protein coupled receptor (GLP-1 receptor) which acts via the adenylyl cyclase (AC) system (Whiting et al.,[7]). CNS and peripheral tissues express receptors of the GLP-1 (Bullock et al., [8]).

The central GLP-1R in cognitive functions is involved, and it is studied (Müller et al. [4]). Several studies have proved that GLP-1R receptor agonism has a promising role to improve cognitive functions (Athauda et al. [9]). Improving associative and spatial learning after activation of CNS GLP-1R signaling was shown by During et al. According to their results, the learning deficit in mice with GLP-1R-deficient was ameliorated after transferring GLP-1R gene to the hippocampus (During et al. [10]). GLP-1R is expressed in the hippocampus of rodents (Rebosio et al. [11]), a part of the brain that incorporates spatial learning and memory (L&M) (Lamsa and Lau [12]). Some features of L&M, including performance in the Morris water maze (MWM) and latency in the passive avoidance test, have improved in rats after intracerebroventricular (ICV) treatment of GLP-1R agonists (Zhou et al. [13]). This effect of GLP-1 on L&M can be diminished by exendin-3 (9-39) which is a GLP-1R antagonist (During et al. [10]). Moreover, the administration of GLP-1R agonists into the hippocampus has led to improve the spatial L&M performances in Alzheimer's disease (Qi et al. [14]; Wang et al. [15]).

It is well accepted that physical activity has beneficial effects on brain activities (Erickson and Kramer, [16]). However, the underlying mechanisms of physical activity which affect the brain largely remain unclear. Previous studies have suggested some mechanisms that, through them, exercise plays a promising role in the brain, such as noradrenergic, serotonergic, and histaminergic neurotransmission (Taati et al., [17]), brain-derived neurotrophic factor (BDNF) (Chen and Russo-Neustadt [18]), insulin-like growth factor I (IGF-1), and vascular endothelial growth factor (VEGF) (Fabel et al. [19]).

Evidence suggests that exercise increases the GLP-1 levels (Ueda et al. [20]). It was indicated that some levels of exercise, from moderate to high-intensity, can enhance GLP-1 levels (Holliday and Blannin [21]). For instance, Ueda et al. have reported that significant increases in GLP-1 plasma levels occurred in terms of exercise (Ueda et al. [22]).

Based on these investigations, we decided to elucidate whether the central GLP-1 receptors have a role in the beneficial effects of exercise on L&M. Simultaneously, we revalidate the previously reported positive effects of GLP-1 on L&M performance.

## 2. Materials and Methods

### 2.1. Animals and Drugs

Thirty-two male Wistar rats (4 months old, an average weight of 300-350 g approximately) were obtained from the Teb Azma animal institute for biological sciences. All animals were maintained in a 12 h light/dark cycle at 21–25°C. Rats were individually housed in standard polycarbonate cages with standard sawdust as bedding. Animals were fed a standard pellet diet which had ad libitum access to water. The current research was authorized by the Lorestan University Ethics Committee (LU. ECRA.2021.7) and conducted in accordance with the National Institutes of Health Guide for the care and use of laboratory animals. Exendin-3 (9-39) (10 *μ*g/rat, 10 *μ*l) (Tocris Co., UK) was mixed in normal saline and injected in intracerebroventricular (ICV) manner (Bell et al. [2]). Rats in saline-treated groups received the same volume of normal saline (10 *μ*l).

### 2.2. Surgical Technique

Anesthesia of animals was performed by intraperitoneal administration of ketamine/xylazine (75 mg/kg-10 mg/kg), and rats were placed on a stereotaxic apparatus (RWD stereotaxic device, serial number: D00751-001, made in China). The method described by Paxinos and Watson (1987) was followed, and a stainless steel guide cannula (gauge 23) was cautiously placed in the right lateral ventricle (Herman and Watson [23]). The stereotaxic coordinates were AP = 0.8, L = 1.5, and V = 3.2 mm. After properly inserting the cannula, it was secured with three stainless steel screws encircling each guiding cannula. Dental methyl methacrylate was then used to secure the cannula and screws to the skull. After surgical treatments, the rats were placed in their respective cages. Prior to conducting tests on rats, a five-day recovery period was accounted for.

### 2.3. Physical Exercise Protocol

After complete recovery, all animals of the exercise group experienced a 17 meters/min exercise for one hour each day, for seven consecutive days, as a mild exercise (Schemmel et al. [24]). To familiarize the animals with the treadmill apparatus and minimize stress, four days before the experiment period, all animals were placed in the treadmill apparatus for 10 min. Treadmill exercise was performed on a rodent treadmill (Tajhiz Gostar Omid Iranian. Co, Karaj, Iran). Running sessions on the treadmill were performed at noon every day. Each exercise began with a 10-minute warm-up (gradual acceleration), and the running pace was raised to 17 meters per minute. The last 10 minutes of the workout consisted of a gradual deceleration. Control group animals were placed on the treadmill for one hour; however, they did not experience an exercise program.

### 2.4. Experimental Design

All animals were randomly divided into four groups (*n* = 8):

Sedentary saline (SED-SAL) group
Sedentary-exendin-3 (SED-exendin) group
3) Treadmill exercise-saline (TE-SAL) group
4) Treadmill exercise-exendin-3 (TE-exendin) group

All rats received the injections just before the exercise manually by hand at a flow rate of 10 *μ*l/minute using a Hamilton syringe (Hamilton 10 *μ*l 701 RN Syringe, USA), daily for seven-day running period. After the last day of running, the animals were tested for L&M tests using Morris water maze (MWM) task.

### 2.5. Morris Water Maze (MWM) Test

We used a standard MWM task which was given in previous reports (Taati et al., [17]). MWM protocol is an acceptable test to evaluate L&M performance (O'Callaghan et al. [25]). The water maze tank was a circular tank with a diameter of 2 m and a depth of 0.4 m that was filled with tap water (23 ± 1°C) on a daily basis. It was placed in a specific room with visual signals on the wall next to the pool. The pool was conceptually separated into four sections, and they were shown by four big marks made by foam for each direction (a star for N, a triangle for S, a rectangle for W, and a circle for E) visible from the surface of tank. An escape square platform (11 cm border), which was considered the target zone, was placed in the northwest quadrant in a permanent position 2 cm under the water surface during the test, except for the last day (measuring the latency). The rats experienced four trials each day for four days. At each trial, the rats were gently placed into the water at a different quadrant chosen randomly. The rat swam to find and locate the hidden platform. If the rats did not find the platform within one minute, they were guided to the platform by hand. A 20 second was considered for rats to rest on the escape platform; therefore, they were taken back to their cages for one minute. A video camera mounted above the center section of the tank recorded the swimming route and the time it took to reach the platform (latency), which was then evaluated using a video tracking path and analysis system. The day following the fourth trial, rats were tested on a probe trial that the escape platform was taken out, and rats swam for one minute. The time that the rats spent in the target zone was recorded for 60 seconds.

### 2.6. Statistical Analysis

The statistical analysis was performed using SPSS software for Windows (SPSS Inc., Chicago, USA, Ver. 25). To examine the interaction among groups and days in 4 levels and to ascertain the significant effects (“group” effect and “day” effect) on escape latency, a two-way ANOVA with repeated measures (days) was utilized for data in the learning phase. Moreover, data regarding memory retention test were analyzed using a two-way ANOVA to specify the significant main effects (“group” effect and “zone” effect) on time spent percentage and the interaction among groups in 4 levels and zones in 2 levels of the target and opposite zones. All analyses were followed by a post hoc Tukey's test regarding the multiple comparison evaluation. All data were expressed as means ± SEM. *P* < 0.5 is considered a statistically significant difference.

## 3. Results

**Figure 1:**
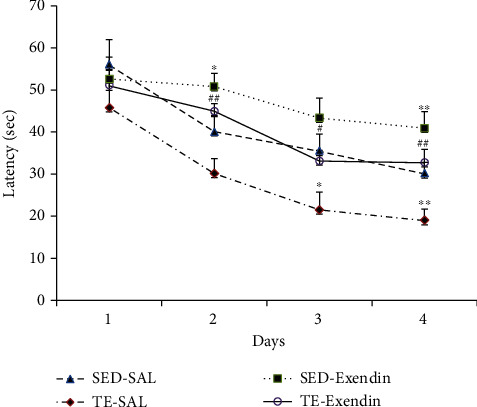
Effects of using the GLP-1 receptors antagonist (exendine-3) during treadmill exercise on learning tested by the MWM task. Data are expressed as the mean ± SEM. ∗ is for *P* < 0.05 and # is for *P* < 0.01.

**Figure 2:**
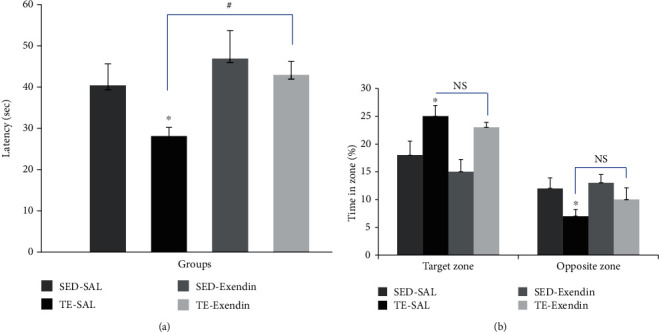
Effect of using the GLP-1-receptors antagonist (exendin-3) during treadmill exercise on memory tested by the MWM task. (a) Mean escape latencies. (b) Meantime spent in the target zone. Data are expressed as the mean ± SEM. ∗ is for *P* < 0.05 and # is for *P* < 0.01.

Acquisition data analysis of all groups is shown in Figure 1 for four days of the trial test. Based on two-way ANOVA test, a significant effect of groups (*F*_3,432_ = 1.707, *P* < 0.01) and days (*F*_3,432_ = 0.804, *P* < 0.01) was revealed on the escape latencies. The results revealed that data regarding escape latencies of treadmill exercise-saline group were remarkably lower than that of sedentary saline group on the third and fourth days of the experiment. The TE-exendin group had a significantly longer escape latency than the TE-SAL group on the second, third, and fourth day (*P* < 0.05). The result of the memory retention test is shown in Figure 2(a). The escape latency of treadmill exercise-saline group was significantly low compared to that of other experimental groups (*P* < 0.05). As shown in Figure 2(b), spent time in the target zone was significantly increased in the treadmill exercise-saline group compared to the sedentary saline group (*P* < 0.05).

## 4. Discussion

In rats, moderate treadmill activity improves L&M performance via the MWM task, and ICV injection of exendin-3 blocks these favorable effects of exercise (9-39). These findings indicate that central GLP-1 receptors may mediate the effects of exercise on L&M. This is the first study we are aware of that has shown that exercise may exert its beneficial effects on cognitive functions via central GLP-1 receptors. This finding that exercise improves L&M confirms our previous study and other research indicating the beneficial effects of exercise on L&M (Liu et al. [26]; Hajisoltani et al. [27]). Treadmill running remains one of the most acceptable samples of physical exercise in rat investigations (Liu et al. [26]).

It is evident that blocking the central GLP-1R by ICV injection of exendin-3 had no significant effect on L&M in sedentary (nonexercised) rats. Since ICV injection of exendin-3 had not shown adverse effects on L&M in normal situations and its use in exercised rats impaired the beneficial effects of exercise on L&M, it is likely to hypothesize that the valuable effects of exercise on L&M may be triggered by the central GLP-1R.

Many reports hypothesized that exercise leads to improve brain function via regulating the GLP-1 release. It is shown that mild and moderate exercise increases GLP-1 concentrations in plasma. For instance, Ueda et al. indicated that an aerobic exercise protocol significantly increased GLP-1 concentration in blood (Ueda et al. [22]). Its physiological processes, however, were unclear. The rise in GLP-1 concentration seen after exercise has been linked to skeletal muscle-derived interleukin-6 or a sciatic nerve afferent route through a humoral pathway in one research (Ellingsgaard et al. [28]). On the other hand, it is proven that the administration of GLP-1R agonists improves L&M (Isacson et al. [29]; Gengler et al. [30]). GLP-1R expression was detected in the brain-specific cellular subtypes, which play a critical role in L&M, such as granule cells of the dentate gyrus in the hippocampus and pyramidal neurons of the CA1 region (Hamilton and Hölscher [31]). Furthermore, since GLP-1's involvement in synaptic plasticity is well understood, it is thought that GLP-1 may have a role in the regulation of many signaling pathways involved in L&M and other synaptic activities (Gault and Hölscher [32]; Simsir et al. [33]). Its glycemic normalization has no influence on these effects (Grieco et al. [34]).

Several mechanisms seem to play a role in exercise-induced enhancement of L&M via GLP-1 receptors. One possibility is an interaction between GLP-1 and hippocampal LTP. Presently, much evidence demonstrates that synaptic plasticity, including LTP, mediates hippocampal-dependent memory (O'Callaghan et al. [25]). On the other hand, it is well accepted that exercise enhances LTP, neurotransmission, cell proliferation, neurogenesis, and expression of genes relating to growth factors in the hippocampus of rats (Cotman and Berchtold [35]). GLP-1 has considerable cognitive-related benefits in the hippocampus, which is an important brain area for L&M, according to data. GLP-1 improves hippocampus LTP production in mice, according to Day et al. (Day et al. [36]). The GLP-1 agonist was discovered to lower potassium channel (Kv4.2) values in the hippocampus, increasing dendritic membrane excitability (Chen et al. [37]). Moreover, Taha et al. (Taha et al. [38]) showed that GLP-1 agonists impede phosphorylation of the mRNA translational factor eEF2 increasing protein synthesis. Hippocampal neurons express Kv4.2 channels, and they act as a regulator in the generation of the backpropagating action potential, which is essential for LTP induction at the hippocampus in physiological situations. Previous research has proven that Kv4.2 inhibits mice from an exhibition of hippocampal LTP production (Chen et al. [37]), and therefore a potential mechanism underlying the early LTP-enhancing effects by GLP-1.

Furthermore, it was emphasized that GLP-1 can enhance BDNF in the brain. It has been clarified in several studies that there is an association between exercise and an increased BDNF level in the brain (Bekinschtein et al. [39]). The role of GLP-1 to inhibit apoptosis, neuronal growth, cell proliferation, and decreasing oxidative damage in the CNS has been recently revealed (Athauda and Foltynie [40]). GLP-1 binding to its receptors which are widely expressed in the brain (Alvarez et al. [41]) increases cAMP and activates the PI3 K (phosphoinositide 3-kinase) signaling pathway, which leads to the activation of AKT (protein kinase B) signaling pathways (Kim et al. [42]). The activation of these pathways results in the production of multiple targets (Athauda and Foltynie [43]), such as GSK-3*β* (glycogen synthase kinase-3*β*), NF*κ*B (nuclear factor-*κ*B), and CREB (cyclic adenosine monophosphate response element-binding protein). Of note, CREB is a crucial player in modulating BDNF production. BDNF was widely studied in cognition, inflammation, and neurogenerative disorders. GLP-1 analogs, such as exenatide and liraglutide, have neuroprotective effects in animal models of Alzheimer's disease (AD) (Athauda and Foltynie [44]).

## 5. Conclusion

In general, we concluded that exercise-induced enhancement of spatial L&M function in rats was prevented by blockade of central GLP-1R using exendin-3. This result indicates that GLP-1 has a significant role to mediate the beneficial effects of physical exercise on cognitive functions.

## Data Availability

All data generated or analyzed during this study are included in this published article.
